# Improvement of ram semen quality by luteolin enrichment during cold preservation

**DOI:** 10.5194/aab-67-123-2024

**Published:** 2024-03-28

**Authors:** Sharif Khozein, Mohsen Eslami, Farhad Farrokhi-Ardabili

**Affiliations:** 1 Department of Theriogenology, Faculty of Veterinary Medicine, Urmia University, Urmia, Iran; 2 Department of Animal Science, Faculty of Agriculture, Urmia University, Urmia, Iran

## Abstract

The present experiment aimed to investigate the probable protective role of luteolin (Lut) in ram spermatozoa kinematics and the oxidative/anti-oxidative/nitrosative status of semen during cold storage. Ejaculates were collected from five Qezel rams twice a week. Ejaculates were pooled, diluted with Tris–egg yolk extender (negative control) or supplemented with 0 (control; received the solvent of luteolin), 4, 8 and 16 µ m Lut. Kinematics parameters, viability and membrane functionality of spermatozoa were assessed. Furthermore, amounts of malondialdehyde (MDA) and anti-oxidant activity (AOA), superoxide dismutase activity (SOD) and total nitrate nitrite (TNN) were evaluated in the medium (diluent) and spermatozoa, separately, at 0, 24, 48 and 72 h after storage at 4 °C. Percentages of forward progressive motility and membrane integrity were significantly higher in 8 and 16 µ m groups compared to control groups at 72 h ( P<0.05 ). No significant differences were observed in viability among groups during the study ( P>0.05 ). Lower MDA contents were observed in medium and spermatozoa of 8 and 16 µ m treated groups compared to controls at 72 h ( P<0.05 ). In addition, higher AOA levels were observed in the medium of Lut-treated groups compared to controls at 48 and 72 h ( P<0.05 ). The activity of SOD was improved by luteolin addition. Luteolin enrichment did not affect TNN amounts. It seems that luteolin (at 8 and 16 µ m) as a flavonoid protects the ram semen by its anti-oxidative properties and by reduction of lipid peroxidation following 48 and 72 h storage.

## 1 Introduction

Semen dilution and preservation allow an increase in the fertile lifespan of spermatozoa, transport it greater distances, and improve the number of inseminations per elite ram (Gillan et al., 2004; Brinsko, 2006). By liquid-cold storage of semen, the fertility of spermatozoa was reported to be good during the first 24 h after collection and storage (Salamon and Maxwell, 2000). However, by extending the storage duration, a sustainable gradual decrease was reported in conception following the insemination of stored semen (Kasimanickam et al., 2007; Munsi et al., 2007). In this situation, spontaneous peroxidation occurred in the ram spermatozoa membrane due to the presence of higher amounts of polyunsaturated fatty acids and more susceptibility of them to peroxidative reaction (Ashrafi et al., 2011). Generation and accumulation of free radicals during chilled preservation reduced the kinematics by alteration of membrane integrity and impairment of acrosome function (Câmara et al., 2011). Therefore, inclusion of protective substances in ram semen extender has been practiced and recommended for combating the destructive effects of oxidative toxicity (Rather et al., 2016; Zadeh-Hashem et al., 2017; Eslami et al., 2019; Rateb, 2018; Nateq et al., 2020; Rateb et al., 2020).

Luteolin [2-[3,4-dihydroxyphenyl]-5,7-dihydroxy-4-chromenone], a phenolic compound, belongs to the flavonoid family and is reported to possess a varied pharmacologic and therapeutic potential (Luo et al., 2019; Yu et al., 2019; Tan et al., 2020; Çetinkaya and Baran 2023; Han et al., 2023). Different studies revealed the anti-oxidative and cell-protective (against oxidative toxicity) roles of luteolin (Qiao et al., 2012; Zhao et al., 2012; Madhesh and Vaiyapuri, 2013; Nazari et al., 2013). However, there is a lack of information on the protective effect of luteolin against the detrimental effects of oxidative toxicity generated during the cold preservation of semen. Therefore, the current study aimed to evaluate the potential protective role of luteolin (Lut, at 2, 4 and 8 µ M concentrations) in ram semen quality during storage at 4 °C. For this purpose, oxidative/anti-oxidative/nitrosative markers of samples were assessed along with the kinematics, viability and membrane integrity of spermatozoa up to 72 h after cold storage.

## 2 Materials and methods

### 2.1 Animals and semen collection

The current study was conducted in the facilities of the Animal Sciences Department in the Faculty of Agriculture, located at Urmia University, Urmia, Iran (Nazloo campus). Five fertile proven rams were used to collect the semen in the presence of an estrus ewe. A total number of 30 semen samples were collected from the five fat-tailed Qezel rams, twice a week, using an artificial vagina during the autumn–winter seasons. Collected samples were immersed in a water bath (37 °C), and the volume and mass motility were recorded immediately. Good-quality samples (volume 0.75–2.5 mL, progressive motility >70 % with mass motility ≥ 4 grade) were pooled (equal volume of each sample) and used for the experiment.

### 2.2 Semen processing and evaluation 

The tris-citric acid-based extender (3.63 g Tris, 0.5 g fructose, 1.99 g citric acid, penicillin G sodium 100 000 IU, streptomycin 100 mg and egg yolk 14 mL; within 100 mL) was prepared (Salamon and Maxwell, 2000) and used in the current experiment. Pooled ejaculates were diluted at $500\times 10^{6}$ spermatozoa per milliliter and allocated for the negative control (no extra addition), control (received dimethyl sulfoxide, the solvent of luteolin) as well as the 4 (Lut 4 µ M), 8 (Lut 8 µ M) and 16 (Lut 16 µ M) µ M luteolin groups. The stock solution of 4 mM Lut was prepared by dissolving it in dimethyl sulfoxide (DMSO) and storing it at - 20 °C until usage. All the groups (except negative control) received an equal volume of DMSO (4 µ L DMSO alone or DMSO + Lut in the 1000 µ L sample). The final percentage of DMSO was 0.4 % in the treated groups. After Lut (72511, CAS no. 491-70-3; product of the USA; Sigma-Aldrich) supplementation, samples were preserved in a refrigerator for up to 72 h. Variables such as kinematics, viability and membrane integrity were assessed at 0, 24, 48 and 72 h. Furthermore, amounts of malondialdehyde (MDA, as the secondary end-product of lipid peroxidation), total anti-oxidant activity (AOA), total nitrate nitrite (TNN; an indicator of nitrosative stress) and superoxide dismutase (SOD) activity were measured within the spermatozoa and the medium, separately, at the studied time points.

### 2.3 Analysis of spermatozoa parameters 

#### 2.3.1 Spermatozoa viability 

Eosin–nigrosin staining was used to evaluate the viable spermatozoa (Evans and Maxwell, 1987). The stained smear was dried, and the percent of viable spermatozoa was reported by counting the colored and non-colored cells under an optic microscope.

#### 2.3.2 Spermatozoa kinematics 

Spermatozoa kinematics were assessed under a phase contrast microscope (Olympus, BX41, Tokyo, Japan) equipped with a stage warmer and a CCD camera (SDC-313B, Samsung Techwin Co., Gyeong, Korea) attached to a computer containing computer-assisted spermatozoa analysis (CASA). To assess the kinematics, the stored samples were extended at 25 × 10 ${}^{6}$ spermatozoa per milliliter and then put on a microscope slide covered with a coverslip, and the capture was performed at 200 × magnification. Total motility (TM), forward progressive motility (FPM), average path velocity (VAP, µ m s ${}^{-1}$ ), straight linear velocity (VSL, µ m s ${}^{-1}$ ), curvilinear velocity (VCL, µ m s ${}^{-1}$ ), straightness (STR = [VSL / VAP] × 100) and linearity (LIN = [VSL / VCL] × 100) were recorded at 0, 24, 48 and 72 h for all the experimental groups.

#### 2.3.3 Membrane functionality of spermatozoa 

Spermatozoa membrane functionality was tested using a hypo-osmotic swelling (HOS) test according to the protocol (Correa and Zavos, 1994). Freshly prepared HOS solution was mixed with samples at a rate of 9:1 and incubated for 1 h at 37 °C.

Spermatozoa with coiled tails were counted under a phase contrast microscope (200 × , Olympus, BX41, Tokyo, Japan) with 200 spermatozoa, and the percentage of spermatozoa with intact plasma membranes was calculated.

By completing the motility, viability and plasma membrane functionality, samples were centrifuged (at 550 g for 10 min) to separate the spermatozoa from the diluent (medium). The recovered pellet was considered spermatozoa, and the supernatant was centrifuged again (3000 g for 30 min) to separate the medium. The spermatozoa pellet was mixed with 0.5 mL phosphate buffer saline and homogenized with a homogenizer (T10 Basic, IKA^®^, Werke GmbH and Co. KG, Staufen, Germany) to release the contents. The separated spermatozoa and medium were stored at - 20 °C until biochemical assessment.

### 2.4 Measurement of biochemical metabolites in the spermatozoa and medium of samples 

The total protein within the spermatozoa and medium of the treated samples was measured according to the standard protocol (Bradford, 1976). In brief, spermatozoa and medium samples were mixed with Bradford reagent, and the test tubes were incubated at laboratory temperature for 10 min. Following recorded absorbance at 595 nm, the protein levels were calculated using the drawn standard plot. The amounts of MDA, TAC, TNN and SOD activity were adjusted with the protein contents of samples.

The MDA amounts were measured according to the previously reported method by Stern et al. (2020). The prepared thiobarbituric reaction was mixed with the sample within a test tube and incubated in boiling water for 15 min. After centrifugation (1000 g for 15 min), the absorbance of the supernatant was recorded at 535 nm using spectrophotometry (Pharmacia, Pharmacia LKB, NOVASPEC II). The MDA amounts of spermatozoa and diluent (medium) samples were expressed in micromol per gram of protein.

The method described by Koracevic et al. (2001) was used to measure the AOA levels within the spermatozoa and medium samples. In brief, after adding the freshly prepared reagents (PBS, acetic acid, Fe-EDTA and H ${}_{2}$ O ${}_{2}$ ), the test tubes were incubated in the water bath for 1 h at 37 °C. In the following, the other reagents (acetic acid and thiobarbituric solution) were added, and the tubes were incubated in boiling water for 10 min. Finally, the produced optical densities of samples were recorded at 532 nm against the blank sample. The AOA levels of spermatozoa and medium were expressed in millimolar per gram of protein.

The Griess reaction described by Green et al. (1982) was used to measure the TNN concentrations. An equal volume of freshly prepared Griess reagent was mixed with the sample within the 96-well plate and incubated in darkness for 10 min. The absorbance of the examined samples was read at 540 nm using an ELISA reader. According to the drawn standard plot, the TNN concentrations were reported in micromol per gram protein in the spermatozoa and medium of samples.

The pyrogallol oxidation method described by Marklund and Marklund (1974) was used to measure the SOD activity within the spermatozoa and medium contents. For this purpose, the prepared tris buffer (PH = 8.5) was mixed with samples, and the absorbance was set to zero at a wavelength of 420 nm using a spectrophotometer (Pharmacia, Pharmacia LKB, NOVASPEC II). In the following, the optical density was recorded at 1.5 and 3.5 min after adding pyrogallol solution to the mixture. The SOD activity was calculated according to the method and reported in micrograms of protein in the tested samples.

### 2.5 Statistical analysis

The effects of the treatment on the motility, viability, membrane functionality and numbers of MDA and AOA parameters were analyzed among the treated groups using one-way ANOVA, followed by a Holm–Šidák post hoc test at each time point. Variable changes among different time points in every group were assessed using repeated-measure ANOVA to reveal time effects. The statistical software that was utilized is SigmaStat (Version 3.5; Chicago, IL). Results are reported as means ± standard error. Mean values were considered to differ when P<0.05 .

## 3 Results 

### Spermatozoa viability, kinematics and functional membrane integrity

The eosin–nigrosin assessment revealed that there were no significant differences in viability between the groups at 0, 24, 48 and 72 h ( P>0.05 ; Table 1). Within-group analysis indicated a lower percentage of viable spermatozoa at 24, 48 and 72 h compared to 0 h in all the experimental groups ( P<0.05 ; Table 1).

**Table 1:** Percentage of total and forward progressive motility, viability and membrane integrity (evaluated by a hypo-osmotic swelling test) of ram spermatozoa following semen supplementation with different concentrations of luteolin (Lut) and storage for various time points at 4 °C.

Parameter	Treatment	Time of storage (h)			
		0	24	48	72
Viable	Negative control	90.75 ± 0.87 ${}^{a}$	80.51 ± 3.35 ${}^{b}$	77.33 ± 1.83 ${}^{\mathrm{bc}}$	71.30 ± 1.39 ${}^{c}$
spermatozoa	Control	89.87 ± 0.48 ${}^{a}$	81.23 ± 2.90 ${}^{b}$	77.11 ± 1.31 ${}^{\mathrm{bc}}$	72.41 ± 1.89 ${}^{c}$
	Lut 4 µ M	87.78 ± 1.77 ${}^{a}$	79.65 ± 2.47 ${}^{b}$	79.37 ± 2.15 ${}^{b}$	75.50 ± 1.51 ${}^{b}$
	Lut 8 µ M	89.57 ± 1.26 ${}^{a}$	81.99 ± 2.27 ${}^{b}$	78.13 ± 1.38 ${}^{\mathrm{bc}}$	74.22 ± 1.86 ${}^{c}$
	Lut 16 µ M	89.40 ± 1.12 ${}^{a}$	78.79 ± 1.48 ${}^{b}$	77.20 ± 0.98 ${}^{b}$	77.81 ± 1.52 ${}^{b}$
Total	Negative control	90.18 ± 1.28 ${}^{a}$	79.16 ± 2.73 ${}^{b}$	75.65 ± 3.55 ${}^{b}$	70.33 ± 1.56 ${}^{b}$
motility	Control	86.49 ± 2.29 ${}^{a}$	77.84 ± 3.04 ${}^{b}$	77.03 ± 3.67 ${}^{b}$	71.60 ± 2.42 ${}^{b}$
	Lut 4 µ M	86.53 ± 1.65 ${}^{a}$	77.01 ± 3.48 ${}^{b}$	75.95 ± 2.08 ${}^{\;b}$	71.95 ± 2.65 ${}^{b}$
	Lut 8 µ M	86.91 ± 2.65 ${}^{a}$	78.79 ± 1.87 ${}^{b}$	75.94 ± 2.72 ${}^{b}$	76.65 ± 1.52 ${}^{b}$
	Lut 16 µ M	88.44 ± 1.08 ${}^{a}$	79.62 ± 2.02 ${}^{b}$	75.55 ± 1.18 ${}^{\mathrm{bc}}$	74.03 ± 1.34 ${}^{c}$
Forward	Negative control	79.49 ± 1.57 ${}^{\mathrm{Aa}}$	49.95 ± 1.84 ${}^{\mathrm{Ab}}$	43.56 ± 1.90 ${}^{\mathrm{Abc}}$	39.45 ± 2.72 ${}^{\mathrm{Ac}}$
progressive	Control	75.78 ± 1.89 ${}^{\mathrm{Aa}}$	51.72 ± 2.56 ${}^{\mathrm{Ab}}$	42.38 ± 1.72 ${}^{\mathrm{Ac}}$	41.79 ± 1.47 ${}^{\mathrm{Ac}}$
motility	Lut 4 µ M	77.32 ± 2.28 ${}^{\mathrm{Aa}}$	54.09 ± 2.26 ${}^{\mathrm{Ab}}$	46.90 ± 2.01 ${}^{\mathrm{ABb}}$	46.35 ± 1.12 ${}^{\mathrm{ABb}}$
	Lut 8 µ M	80.71 ± 1.44 ${}^{\mathrm{Aa}}$	56.60 ± 0.95 ${}^{\mathrm{Ab}}$	51.38 ± 2.17 ${}^{\mathrm{Bbc}}$	49.54 ± 1.28 ${}^{\mathrm{Bc}}$
	Lut 16 µ M	76.42 ± 1.63 ${}^{\mathrm{Aa}}$	56.10 ± 1.84 ${}^{\mathrm{Ab}}$	50.21 ± 1.79 ${}^{\mathrm{Bb}}$	50.07 ± 1.82 ${}^{\mathrm{Bb}}$
Membrane	Negative control	80.03 ± 1.17 ${}^{\mathrm{Aa}}$	73.22 ± 1.42 ${}^{\mathrm{Ab}}$	67.24 ± 2.15 ${}^{\mathrm{Abc}}$	62.48 ± 1.84 ${}^{\mathrm{Ac}}$
integrity of	Control	81.49 ± 1.54 ${}^{\mathrm{Aa}}$	74.73 ± 1.72 ${}^{\mathrm{Ab}}$	70.38 ± 2.10 ${}^{\mathrm{Ab}}$	63.57 ± 1.58 ${}^{\mathrm{ABc}}$
spermatozoa	Lut 4 µ M	82.18 ± 1.51 ${}^{\mathrm{Aa}}$	76.97 ± 2.32 ${}^{\mathrm{Ab}}$	73.45 ± 1.75 ${}^{\mathrm{Ab}}$	67.45 ± 2.10 ${}^{\mathrm{ABc}}$
	Lut 8 µ M	82.07 ± 1.87 ${}^{\mathrm{Aa}}$	78.97 ± 2.62 ${}^{\mathrm{Aab}}$	72.57 ± 2.28 ${}^{\mathrm{Abc}}$	70.48 ± 1.89 ${}^{\mathrm{Bc}}$
	Lut 16 µ M	84.12 ± 1.41 ${}^{\mathrm{Aa}}$	78.84 ± 2.49 ${}^{\mathrm{Aab}}$	72.88 ± 2.61 ${}^{\mathrm{Abc}}$	69.02 ± 1.12 ${}^{\mathrm{Bc}}$

${}^{A,B}$ Values with different superscripts indicate a difference ( P<0.05 ) between the groups at each time point. ${}^{a,b,c}$ Values with different superscripts indicate a difference ( P<0.05 ) between the different time points in each group.

Total motility did not differ between the experimental groups at 0, 24, 48 and 72 h ( P<0.05 ; Table 1). The percentage of TM was reduced at 24 h compared to 0 h in all the groups, and the differences continued for 48 and 72 time points ( P<0.05 ; Table 1). A higher percentage of FPM was observed in the 8 and 16 µ M luteolin-treated groups compared to the control groups at 48 and 72 h ( P<0.05 ; Table 1).

A higher percentage of spermatozoa with intact membranes was detected in the Lut 8 and 16 µ M treated groups compared to the controls at 72 h ( P<0.05 ; Table 1). Repeated-measure analysis revealed that the percentage of intact-membrane spermatozoa was reduced at 48 and 72 h compared to 0 h in all the groups ( P<0.05 ; Table 1).

Higher VAP (at 48 and 72 h), VSL (at 72 h) and VCL (at 48 and 72 h) were recorded for the 8 and 16 µ M luteolin-treated groups compared to the control groups ( P<0.05 ; Table 2). There were no significant differences in the STR and LIN variables between the groups during the study ( P>0.05 ; Table 2).

Amounts of MDA within spermatozoa did not differ between the groups at 0, 24 and 48 h ( P>0.05 ; Table 3), while lower MDA levels were recorded in the Lut 8 and 16 groups than controls at 72 h of storage ( P<0.05 ; Table 3). Within-group analysis indicated higher amounts of MDA levels at 24, 48 and 72 h compared to 0 h in all the groups.

**Table 2:** Kinematics variables of ram spermatozoa analyzed using the CASA system following semen supplementation with different concentrations of Lut and storage for various time points at 4 °C.

Parameter	Treatment	Time of storage (h)			
		0	24	48	72
VAP ( µ m s ${}^{-1}$ )	Negative control	38.72 ± 1.33 ${}^{\mathrm{Aa}}$	32.11 ± 1.46 ${}^{\mathrm{Aab}}$	25.16 ± 1.88 ${}^{\mathrm{Ab}}$	21.01 ± 1.31 ${}^{\mathrm{Ac}}$
	Control	39.02 ± 1.60 ${}^{\mathrm{Aa}}$	33.21 ± 2.29 ${}^{\mathrm{Aab}}$	27.28 ± 1.62 ${}^{\mathrm{ABb}}$	23.11 ± 1.42 ${}^{\mathrm{Ab}}$
	Lut 4 µ M	39.93 ± 1.16 ${}^{\mathrm{Aa}}$	33.15 ± 1.50 ${}^{\mathrm{Aab}}$	29.06 ± 1.08 ${}^{\mathrm{ABbc}}$	24.21 ± 1.20 ${}^{\mathrm{ABc}}$
	Lut 8 µ M	40.42 ± 2.69 ${}^{\mathrm{Aa}}$	34.40 ± 1.78 ${}^{\mathrm{Aab}}$	31.87 ± 1.66 ${}^{\mathrm{Bbc}}$	27.19 ± 1.44 ${}^{\mathrm{Bc}}$
	Lut 16 µ M	40.30 ± 1.20 ${}^{\mathrm{Aa}}$	35.14 ± 1.84 ${}^{\mathrm{Aab}}$	30.99 ± 1.19 ${}^{\mathrm{Bbc}}$	27.37 ± 1.57 ${}^{\mathrm{Bc}}$
VSL ( µ m s ${}^{-1}$ )	Negative control	26.21 ± 1.33 ${}^{\mathrm{Aa}}$	21.31 ± 1.04 ${}^{\mathrm{Ab}\;}$	18.44 ± 0.67 ${}^{\mathrm{Abc}}$	14.18 ± 0.64 ${}^{\mathrm{Ac}}$
	Control	24.88 ± 1.90 ${}^{\mathrm{Aa}}$	21.91 ± 0.89 ${}^{\mathrm{Aab}}$	18.48 ± 1.08 ${}^{\mathrm{Abc}}$	13.27 ± 0.90 ${}^{\mathrm{Ac}}$
	Lut 4 µ M	24.29 ± 1.77 ${}^{\mathrm{Aa}}$	20.69 ± 1.23 ${}^{\mathrm{Aab}}$	20.21 ± 1.09 ${}^{\mathrm{Aab}}$	16.06 ± 0.61 ${}^{\mathrm{ABb}}$
	Lut 8 µ M	25.33 ± 1.77 ${}^{\mathrm{Aa}}$	22.78 ± 0.86 ${}^{\mathrm{Aa}}$	20.71 ± 1.33 ${}^{\mathrm{Aa}}$	19.28 ± 0.77 ${}^{\mathrm{Ba}}$
	Lut 16 µ M	26.43 ± 1.50 ${}^{\mathrm{Aa}}$	22.66 ± 1.21 ${}^{\mathrm{Aab}\;}$	19.60 ± 0.96 ${}^{\mathrm{Aab}}$	18.33 ± 0.94 ${}^{\mathrm{Bb}}$
VCL ( µ m s ${}^{-1}$ )	Negative control	122.83 ± 3.13 ${}^{\mathrm{Aa}}$	93.82 ± 1.55 ${}^{\mathrm{Ab}}$	77.31 ± 2.88 ${}^{\mathrm{Ac}}$	65.31 ± 2.33 ${}^{\mathrm{Ad}}$
	Control	118.24 ± 3.41 ${}^{\mathrm{Aa}}$	98.83 ± 3.59 ${}^{\mathrm{Aab}}$	80.26 ± 2.52 ${}^{\mathrm{Ab}}$	68.37 ± 2.09 ${}^{\mathrm{Ac}}$
	Lut 4 µ M	120.29 ± 3.37 ${}^{\mathrm{Aa}}$	100.25 ± 4.02 ${}^{\mathrm{Ab}}$	88.05 ± 3.11 ${}^{\mathrm{ABc}}$	77.71 ± 1.99 ${}^{\mathrm{Bc}}$
	Lut 8 µ M	122.56 ± 3.30 ${}^{\mathrm{Aa}}$	103.74 ± 3.17 ${}^{\mathrm{Ab}}$	93.26 ± 3.59 ${}^{\mathrm{Bbc}}$	88.71 ± 2.21 ${}^{\mathrm{Bc}}$
	Lut 16 µ M	119.99 ± 5.67 ${}^{\mathrm{Aa}}$	101.50 ± 2.34 ${}^{\mathrm{Ab}}$	91.34 ± 3.41 ${}^{\mathrm{Bbc}}$	85.67 ± 2.17 ${}^{\mathrm{Bc}}$
STR (%)	Negative control	74.35 ± 1.80 ${}^{\mathrm{Aa}\;}$	72.12 ± 2.02 ${}^{\mathrm{Aa}}$	71.59 ± 1.70 ${}^{\mathrm{Aa}}$	71.20 ± 1.53 ${}^{\mathrm{Aa}\;}$
	Control	72.17 ± 1.93 ${}^{\mathrm{Aa}}$	73.38 ± 1.50 ${}^{\mathrm{Aa}}$	70.38 ± 2.28 ${}^{\mathrm{Aa}}$	71.44 ± 1.51 ${}^{\mathrm{Aa}}$
	Lut 4 µ M	71.94 ± 2.03 ${}^{\mathrm{Aa}}$	71.78 ± 2.31 ${}^{\mathrm{Aa}}$	72.48 ± 1.71 ${}^{\mathrm{Aa}}$	71.96 ± 1.34 ${}^{\mathrm{Aa}}$
	Lut 8 µ M	74.17 ± 1.77 ${}^{\mathrm{Aa}}$	73.44 ± 1.04 ${}^{\mathrm{Aa}}$	71.59 ± 1.55 ${}^{\mathrm{Aa}}$	72.11 ± 1.84 ${}^{\mathrm{Aa}}$
	Lut 16 µ M	73.27 ± 1.44 ${}^{\mathrm{Aa}}$	73.19 ± 1.11 ${}^{\mathrm{Aa}}$	73.86 ± 2.20 ${}^{\mathrm{Aa}}$	73.20 ± 2.05 ${}^{\mathrm{Aa}}$
LIN (%)	Negative control	27.11 ± 1.90 ${}^{\mathrm{Aa}}$	27.33 ± 2.19 ${}^{\mathrm{Aa}}$	26.279 ± 1.11 ${}^{\mathrm{Aa}}$	27.47 ± 1.19 ${}^{\mathrm{Aa}\;}$
	Control	28.10 ± 2.58 ${}^{\mathrm{Aa}}$	27.80 ± 2.20 ${}^{\mathrm{Aa}}$	27.10 ± 1.79 ${}^{\mathrm{Aa}}$	28.17 ± 0.93 ${}^{\mathrm{Aa}}$
	Lut 4 µ M	28.39 ± 1.89 ${}^{\mathrm{Aa}}$	28.38 ± 1.88 ${}^{\mathrm{Aa}}$	28.64 ± 1.91 ${}^{\mathrm{Aa}}$	27.78 ± 2.13 ${}^{\mathrm{Aa}}$
	Lut 8 µ M	29.81 ± 1.55 ${}^{\mathrm{Aa}}$	28.19 ± 1.69 ${}^{\mathrm{Aa}}$	27.15 ± 1.16 ${}^{\mathrm{Aa}}$	27.65 ± 1.52 ${}^{\mathrm{Aa}}$
	Lut 16 µ M	28.51 ± 2.38 ${}^{\mathrm{Aa}}$	27.58 ± 1.06 ${}^{\mathrm{Aa}}$	26.80 ± 1.08 ${}^{\mathrm{Aa}}$	27.17 ± 1.04 ${}^{\mathrm{Aa}}$

${}^{A,B}$ Values with different superscripts indicate a difference ( P<0.05 ) between the groups at each time point. ${}^{a,b,c}$ Values with different superscripts indicate a difference ( P<0.05 ) between the different time points in each group.

**Table 3:** Amounts of malondialdehyde (MDA; nmol g ${}^{-1}$ protein) in medium and spermatozoa of rams following supplementation with different levels of Lut and storage for various time points at 4 °C.

Parameter	Treatment	Time of storage (h)			
		0	24	48	72
MDA in	Negative control	285.14 ± 10.65 ${}^{\mathrm{Aa}}$	335.32 ± 18.13 ${}^{\mathrm{Aab}}$	390.11 ± 17.56 ${}^{\mathrm{Abc}}$	409.87 ± 17.05 ${}^{\mathrm{Ac}}$
medium	Control	275.87 ± 19.41 ${}^{\mathrm{Aa}}$	337. 65 ± 16.76 ${}^{\mathrm{Aab}}$	388.90 ± 18.65 ${}^{\mathrm{Ab}}$	412.55 ± 17.70 ${}^{\mathrm{Ab}}$
	Lut 4 µ M	294.32 ± 14.29 ${}^{\mathrm{Aa}}$	317.44 ± 5.52 ${}^{\mathrm{Aa}}$	334.93 ± 11.54 ${}^{\mathrm{Aab}}$	379.72 ± 19.68 ${}^{\mathrm{ABb}}$
	Lut 8 µ M	288.67 ± 19.45 ${}^{\mathrm{Aa}}$	301.42 ± 10.07 ${}^{\mathrm{Aab}}$	316.87 ± 26.76 ${}^{\mathrm{Aab}}$	344.23 ± 12.87 ${}^{\mathrm{Bb}}$
	Lut 16 µ M	277.83 ± 13.49 ${}^{\mathrm{Aa}}$	299.55 ± 17.54 ${}^{\mathrm{Aab}}$	319.34 ± 16.39 ${}^{\mathrm{Aab}}$	352.22 ± 6.90 ${}^{\mathrm{Bb}}$
MDA in	Negative control	88.80 ± 5.19 ${}^{\mathrm{Aa}}$	111.54 ± 11.32 ${}^{\mathrm{Ab}}$	120.32 ± 12.89 ${}^{\mathrm{Abc}}$	248.21 ± 28.76 ${}^{\mathrm{Ac}}$
spermatozoa	Control	83.44 ± 7.41 ${}^{\mathrm{Aa}}$	95.44 ± 6.68 ${}^{\mathrm{Ab}}$	113.43 ± 14.54 ${}^{\mathrm{Ac}}$	227.43 ± 9.60 ${}^{\mathrm{Ac}}$
	Lut 4 µ M	90.07 ± 7.38 ${}^{\mathrm{Aa}}$	99.49 ± 6.79 ${}^{\mathrm{Ab}}$	117.21 ± 11.65 ${}^{\mathrm{Ab}}$	187.63 ± 11.98 ${}^{\mathrm{ABb}}$
	Lut 8 µ M	105.44 ± 7.93 ${}^{\mathrm{Aa}}$	106.65 ± 7.08 ${}^{\mathrm{Ab}}$	127.87 ± 9.30 ${}^{\mathrm{Abc}}$	147.79 ± 25.43 ${}^{\mathrm{Bc}}$
	Lut 16 µ M	98.40 ± 9.03 ${}^{\mathrm{Aa}}$	95.54 ± 11.14 ${}^{\mathrm{Ab}}$	126.42 ± 7.29 ${}^{\mathrm{Ab}}$	154.52 ± 9.90 ${}^{\mathrm{Bb}}$

${}^{A,B,C}$ Values with different superscripts indicate a difference ( P<0.05 ) between the groups at each time point. ${}^{a,b,c,d}$ Values with different superscripts indicate significant differences ( P<0.05 ) between the different time points in each group.

The MDA levels in the medium of the Lut 8 and 16 groups were lower compared to the controls at 72 h (Table 3). Furthermore, MDA levels were greater at 72 h compared to 0 h in all the groups ( P<0.05 ; Table 3).

Amounts of AOA were greater in the medium of luteolin-treated groups compared to the negative control group at 0 h and compared to controls at 24, 48 and 72 h of storage ( P<0.05 ; Table 4). Time series analysis revealed no significant difference between the time points within the treated groups ( P>0.05 ; Table 4).

The AOA levels within spermatozoa did not differ between the treated groups at the studied time points ( P>0.05 ; Table 4). However, within-group analysis indicated higher AOA levels of the control groups at 72 h compared to 0 h ( P<0.05 ; Table 4).

Analysis indicated that SOD activity was greater in the medium and within the spermatozoa of the Lut 8 and 16 groups compared to the controls at 48 and 72 h ( P<0.05 ; Table 5).

The amount of TNN did not differ between the treated groups in the medium and within the spermatozoa at the studied time points ( P>0.05 ; Table 6).

**Table 4:** Total anti-oxidant activity (AOA; mmol g ${}^{-1}$ protein) in medium and spermatozoa of rams following supplementation with different levels of Lut and storage for various time points at 4 °C.

Parameter	Treatment	Time of storage (h)			
		0	24	48	72
Total AOA in	Negative control	3.76 ± 0.23 ${}^{A}$	3.37 ± 0.16 ${}^{A}$	3.47 ± 0.17 ${}^{A}$	3.52 ± 0.28 ${}^{A}$
medium	Control	4.27 ± 0.04 ${}^{\mathrm{AB}}$	4.03 ± 0.08 ${}^{B}$	3.85 ± 0.20 ${}^{A}$	3.93 ± 0.14 ${}^{A}$
	Lut 4 µ M	4.76 ± 0.10 ${}^{B}$	4.76 ± 0.08 ${}^{C}$	4.65 ± 0.14 ${}^{B}$	4.76 ± 0.05 ${}^{B}$
	Lut 8 µ M	4.39 ± 0.17 ${}^{B}$	4.68 ± 0.13 ${}^{C}$	4.48 ± 0.05 ${}^{B}$	4.59 ± 0.11 ${}^{B}$
	Lut 16 µ M	4.56 ± 0.14 ${}^{B}$	4.70 ± 0.17 ${}^{C}$	4.76 ± 0.14 ${}^{B}$	4.92 ± 0.01 ${}^{B}$
Total AOA in	Negative control	1.06 ± 0.05 ${}^{a}$	1.36 ± 0.03 ${}^{\mathrm{ab}}$	1.45 ± 0.12 ${}^{b}$	1.55 ± 0.01 ${}^{b}$
spermatozoa	Control	1.25 ± 0.14 ${}^{\mathrm{ab}}$	1.10 ± 0.05 ${}^{a}$	1.24 ± 0.16 ${}^{\mathrm{ab}}$	1.48 ± 0.15 ${}^{b}$
	Lut 4 µ M	1.23 ± 0.03 ${}^{a}$	1.41 ± 0.04 ${}^{a}$	1.38 ± 0.08 ${}^{a}$	1.54 ± 0.10 ${}^{a}$
	Lut 8 µ M	1.51 ± 0.12 ${}^{a}$	1.47 ± 0.16 ${}^{a}$	1.75 ± 0.08 ${}^{a}$	1.68 ± 0.06 ${}^{a}$
	Lut 16 µ M	1.45 ± 0.12 ${}^{a}$	1.32 ± 0.09 ${}^{a}$	1.58 ± 0.14 ${}^{a}$	1.73 ± 0.06 ${}^{a}$

${}^{A,B}$ Values with different superscripts indicate a difference ( P<0.05 ) between the groups at each time point for each parameter. ${}^{a,b}$ Values with different superscripts indicate a significant difference ( P<0.05 ) between the different time points in each group (there were no significant differences between the different time points in amounts of AOA in the medium).

**Table 5:** Superoxide dismutase activity (SOD; mg ${}^{-1}$ protein) within the medium and spermatozoa of rams following supplementation with different levels of Lut and storage for various time points at 4 °C.

Parameter	Treatment	Time of storage (h)			
		0	24	48	72
SOD activity in	Negative control	15.51 ± 1.67 ${}^{\mathrm{Aa}}$	16.11 ± 1.15 ${}^{\mathrm{Aa}}$	15.46 ± 0.38 ${}^{\mathrm{Aa}}$	14.65 ± 1.05 ${}^{\mathrm{Aa}}$
the medium	Control	17.20 ± 1.01 ${}^{\mathrm{Aa}}$	18.19 ± 1.59 ${}^{\mathrm{Aa}}$	16.91 ± 0.21 ${}^{\mathrm{Aa}}$	16.50 ± 1.01 ${}^{\mathrm{ABa}}$
	Lut 4 µ M	16.91 ± 1.20 ${}^{\mathrm{Aa}}$	18.89 ± 0.77 ${}^{\mathrm{Aa}}$	19.11 ± 0.38 ${}^{\mathrm{ABa}}$	19.78 ± 0.68 ${}^{\mathrm{BCa}}$
	Lut 8 µ M	15.71 ± 0.89 ${}^{\mathrm{Aa}}$	18.33 ± 1.42 ${}^{\mathrm{Aab}}$	21.88 ± 0.34 ${}^{\mathrm{Bbc}}$	25.20 ± 0.89 ${}^{\mathrm{Cc}}$
	Lut 16 µ M	15.16 ± 1.12 ${}^{\mathrm{Aa}}$	17.58 ± 1.12 ${}^{\mathrm{Aab}}$	22.73 ± 0.27 ${}^{\mathrm{Bb}}$	24.17 ± 0.94 ${}^{\mathrm{Cb}}$
SOD activity	Negative control	5.11 ± 0.31 ${}^{\mathrm{Aa}}$	4.75 ± 0.39 ${}^{\mathrm{Aab}}$	4.41 ± 0.56 ${}^{\mathrm{Abc}}$	4.21 ± 0.71 ${}^{\mathrm{Ac}}$
within the	Control	5.08 ± 0.56 ${}^{\mathrm{Aa}}$	4.58 ± 0.26 ${}^{\mathrm{Aab}}$	4.63 ± 0.42 ${}^{\mathrm{Aab}}$	4.43 ± 0.58 ${}^{\mathrm{Ab}}$
spermatozoa	Lut 4 µ M	5.71 ± 0.32 ${}^{\mathrm{Aa}}$	5.21 ± 0.38 ${}^{\mathrm{Aab}}$	4.93 ± 0.31 ${}^{\mathrm{ABb}}$	5.20 ± 0.42 ${}^{\mathrm{ABab}}$
	Lut 8 µ M	5.37 ± 0.76 ${}^{\mathrm{Aa}}$	5.55 ± 0.51 ${}^{\mathrm{Aa}}$	5.87 ± 0.39 ${}^{\mathrm{Ba}}$	5.79 ± 0.38 ${}^{\mathrm{Ba}}$
	Lut 16 µ M	5.46 ± 0.42 ${}^{\mathrm{Aa}}$	5.41 ± 0.44 ${}^{\mathrm{Aa}}$	5.64 ± 0.27 ${}^{\mathrm{Ba}}$	5.91 ± 0.51 ${}^{\mathrm{Ba}}$

${}^{A,B,C}$ Values with different superscripts indicate a difference ( P<0.05 ) between the groups at each time point. ${}^{a,b,c}$ Values with different superscripts indicate significant differences ( P<0.05 ) between the different time points in each group.

## 4 Discussion

The current study showed that luteolin supplementation (at the 8 and 16 µ M levels) increased the quality of liquid-cold-stored ram semen samples by increasing anti-oxidant capacity (total and enzymatic) and reducing spermatozoa membrane peroxidative reaction. However, the nitrosative reaction was not influenced by luteolin addition. To the best of our knowledge, our current study is the first finding concerning the protective role of luteolin in ram semen during cold preservation.

In the present study, luteolin enrichment resulted in a non-significant increase in the viability of ram spermatozoa. At the same time, progressive motility and other kinematics variables such as VAP, VSL and VCL were improved by 8 and 16 µ M luteolin supplementation at 48 and 72 h. Motility is the most important indicator of fertilization capacity (İnanç et al., 2022). On the other hand, VCL is the most significant and independent parameter of CASA, which highly correlates with the fertility of spermatozoa (Larsen et al., 2000). Suppressive roles of luteolin against initiation and development of apoptotic signaling (neural, renal, hepatic and cardiac) by upregulating the anti-apoptotic protein (Bcl-2) and downregulating pro-apoptotic proteins (caspase-3 and Bax) have been documented by in vivo experiments (Wei et al., 2018; Al-Megrin et al., 2019; Albarakati et al., 2020; Baty et al., 2020). However, viability was not statistically improved by luteolin administration in our experiment. In accordance with our experiment, another study revealed that luteolin was ineffective in anti-apoptotic pathways (Choi et al., 2003). Why the luteolin does not express an anti-apoptotic role in some experiments is unknown to the authors. Testes and epididymis weights were recovered by co-treatment of luteolin + doxorubicin (an anti-cancer drug generating reactive oxygen and nitrogen species) in rat models (Owumi et al., 2020). Furthermore, the total number of spermatozoa, their motility and their viability were significantly improved by luteolin administration (Owumi et al., 2020). An in vivo research model approved the protective role of luteolin against electromagnetic field deleterious effects by increasing the number of leydig cells and the percentage of normal spermatozoa and spermatids (Yahyazadeh and Altunkaynak, 2019). A recent study showed that luteolin supplementation improved the total and progressive motility of frozen–thawed rabbit semen samples, while the viability was not affected (Akarsu et al., 2023), which is compatible with our results.

**Table 6:** Amounts of total nitrate nitrite (TNN; nmol g ${}^{-1}$ protein) in medium and spermatozoa of rams following supplementation with different levels of Lut and storage for various time points at 4 °C.

Parameter	Treatment	Time of storage (h)			
		0	24	48	72
TNN in the	Negative control	214 ± 9.21 ${}^{\mathrm{Aa}}$	228 ± 8.22 ${}^{\mathrm{Aab}}$	230 ± 9.17 ${}^{\mathrm{Aab}}$	242 ± 6.38 ${}^{\mathrm{Ab}}$
medium	Control	229 ± 11.21 ${}^{\mathrm{Aa}}$	235 ± 9.17 ${}^{\mathrm{Aa}}$	233 ± 7.15 ${}^{\mathrm{Aa}}$	236 ± 8.14 ${}^{\mathrm{Aa}}$
	Lut 4 µ M	233 ± 10.33 ${}^{\mathrm{Aa}}$	240 ± 6.19 ${}^{\mathrm{Aa}}$	246 ± 8.19 ${}^{\mathrm{Aa}}$	241 ± 7.75 ${}^{\mathrm{Aa}}$
	Lut 8 µ M	221 ± 8.17 ${}^{\mathrm{Aa}}$	237 ± 8.22 ${}^{\mathrm{Aa}}$	232 ± 5.16 ${}^{\mathrm{Aa}}$	255 ± 7.19 ${}^{\mathrm{Aa}}$
	Lut 16 µ M	218 ± 9.25 ${}^{\mathrm{Aa}}$	230 ± 9.28 ${}^{\mathrm{Aa}}$	235 ± 7.12 ${}^{\mathrm{Aa}}$	233 ± 5.10 ${}^{\mathrm{Aa}}$
TNN in the	Negative control	53.06 ± 2.34 ${}^{\mathrm{Aa}}$	58.27 ± 3.13 ${}^{\mathrm{Aab}}$	57.45 ± 2.88 ${}^{\mathrm{Aab}}$	63.55 ± 3.21 ${}^{\mathrm{Ab}}$
spermatozoa	Control	58.25 ± 4.21 ${}^{\mathrm{Aa}}$	55.81 ± 2.45 ${}^{\mathrm{Aa}}$	61.45 ± 3.16 ${}^{\mathrm{Aa}}$	60.34 ± 3.65 ${}^{\mathrm{Aa}}$
	Lut 4 µ M	55.23 ± 5.33 ${}^{\mathrm{Aa}}$	56.79 ± 2.34 ${}^{\mathrm{Aa}}$	62.38 ± 3.88 ${}^{\mathrm{Aa}}$	65.34 ± 4.10 ${}^{\mathrm{Aa}}$
	Lut 8 µ M	52.51 ± 2.13 ${}^{\mathrm{Aa}}$	59.56 ± 4.36 ${}^{\mathrm{Aa}}$	58.55 ± 3.35 ${}^{\mathrm{Aa}}$	69.46 ± 4.38 ${}^{\mathrm{Ab}}$
	Lut 16 µ M	56.45 ± 3.18 ${}^{\mathrm{Aa}}$	60.43 ± 4.35 ${}^{\mathrm{Aab}}$	63.58 ± 4.16 ${}^{\mathrm{Aab}}$	68.15 ± 4.22 ${}^{\mathrm{Ab}}$

There were no significant differences between the treated groups at each time point for TNN in the medium and spermatozoa. ${}^{a,b}$ Values with different superscripts indicate significant differences ( P<0.05 ) between the different time points in each group.

Decreasing oxidants and their products and increasing anti-oxidant enzyme activity by upregulation of the nuclear factor erythroid-2-related factor 2 (Nrf2)/heme oxygenase-1 (HO-1) and anti-oxidant response element (ARE) are the known protective mechanisms of luteolin (Yang et al., 2016; Kang et al., 2017; Alekhya et al., 2019; Albarakati et al., 2020; Cho et al., 2020). The Nrf2 is a transcriptional regulator that controls the expression of anti-oxidants and cytoprotective proteins against oxidant and oxidative substances (Almeer et al., 2018). Reactive oxygen species cause translocation of Nrf2 to the nucleus and enhance the ARE to generate enzymatic anti-oxidants such as catalase, SOD, nitrite oxidase and HO-1 (Jang et al., 2022). Furthermore, luteolin has been known to raise the level of reduced glutathione and upregulate the expression of glutathione synthetase, which is a potent anti-oxidant (Raj Rai et al., 2021). The current study indicated that luteolin (8 and 16 µ M) was able to improve the anti-oxidative levels (total and SOD activity; 24–72 h), reduce the peroxidative reaction (at 72 h), and finally improve the quality of ram semen during cold preservation. Many studies indicated the protective role of luteolin against oxidative agents or toxic substances by in vivo and in vitro experiments. In this regard, research revealed the ameliorative role of luteolin even against cancerous agents by upregulation of enzymatic anti-oxidants (catalase, SOD) and reduction of lipid peroxidation indices in tissues and samples (Zhang et al., 2016). The protective role of luteolin against electromagnetic field deleterious effects by increasing serum levels of SOD activity, testes weight and testosterone levels compared to the exposed group has been documented (Yahyazadeh and Altunkaynak, 2019). The detrimental effects of excessive production of reactive oxygen species (ROS) on spermatozoa motility by reducing axonemal protein phosphorylation and spermatozoa immobilization have been documented (De Lamirande and Gagnon, 1995). Malondialdehyde is the main aldehyde product due to the degradation of lipid hydroperoxides, and it can cross-link the DNA and proteins (Esterbauer et al., 1991). Due to higher amounts of polyunsaturated fatty acids and a lower anti-oxidative capacity, the ram spermatozoa are very sensitive to oxidative/nitrosative toxicity following cold-liquid storage (Alvarez et al., 1987; Griveau et al., 1995). Lipid peroxidation of spermatozoa membranes resulted in irreversible motility loss, structural damage to the membrane, inhibition of fructolysis and respiration and finally loss of viability in spermatozoa (Dunnet, 1980; Maxwell and Watson, 1996). A recent study showed that luteolin was remarkably able to modulate the detrimental effects of plumbism reproductive toxicity through reduction of testicular lipid peroxidation and nitric oxide levels (nitrosative reaction) as well as augmentation of enzymatic and non-enzymatic anti-oxidant levels of rat testes (Al-Megrin et al., 2020). Another experiment showed that luteolin administration restored the activity of anti-oxidative enzymes (GPx, catalase, SOD) in the testes and epididymis tissues compared to the doxorubicin control group (Owumi et al., 2020). Moreover, reactive oxygen or nitrogen species and the lipid peroxidative index of reproductive tissues were lower through luteolin co-administration with doxorubicin in the rat model (Owumi et al., 2020). Another study indicated lower amounts of ROS in frozen–thawed rabbit semen samples compared to the control sample and finally resulted in an improvement in semen quality (Akarsu et al., 2023). In the current experiment, Nrf2/HO-1 was not assessed. However, AOA and SOD amounts verified the role of luteolin in the upregulation of enzymatic and non-enzymatic anti-oxidants and reduction of MDA as the main peroxidative indicator of the spermatozoa membrane during cold preservation.

## 5 Conclusions

In conclusion, the current study showed the protective role of luteolin in the preservation of ram spermatozoa membranes and motility during liquid-cold storage. Furthermore, luteolin was able to reduce the lipid peroxidative index and increase the enzymatic and non-enzymatic anti-oxidants. These findings let us propose the luteolin enrichment at effective doses during chilled storage of ram semen. The role of luteolin against freezing or thawing adverse effects will be investigated in the future on semen samples of animals.

## Data Availability

The data used and analyzed during this study are available from the corresponding author upon reasonable request.
